# The Role of the Mannheim Peritonitis Index for Predicting Outcomes in Patients With Perforation Peritonitis in a Rural Hospital in India

**DOI:** 10.7759/cureus.36620

**Published:** 2023-03-24

**Authors:** Harshal Ramteke, Swati G Deshpande, Rohini Bhoyar

**Affiliations:** 1 General Surgery, Jawaharlal Nehru Medical College, Datta Meghe Institute of Higher Education and Research, Wardha, IND; 2 Obstetrics and Gynaecology, NKP Salve Institute of Medical Sciences, Nagpur, IND

**Keywords:** mortality, prognosis, mannheim peritonitis index, secondary peritonitis, hollow viscus perforation

## Abstract

Introduction

Acute secondary peritonitis due to hollow viscus perforation is a life-threatening surgical condition with significant morbidity and mortality, depending on the severity with outcomes that differ in the Western and developing world. Various scoring systems have been developed to assess the severity and its relation to morbidity and mortality. We conducted this study to evaluate the role of the Mannheim peritonitis index (MPI) in predicting outcomes in perforation peritonitis patients in a rural hospital in India.

Materials and methods

A prospective study of 50 patients with hollow viscus perforation with secondary peritonitis presented to the emergency department, Acharya Vinoba Bhave Rural Hospital, Sawangi (Meghe), Wardha, from 2016 to 2020. Each operated patient was scored according to the MPI to predict mortality.

Results

The majority of the patients were discharged uneventfully and about 16% (8/50) of the patients expired. The patients with an MPI score of more than 29 had maximum mortality of 62.5%. Mortality was seen in 37.5% of the patients with MPI scores between 21 and 29, whereas no mortality was recorded in patients with an MPI score of 21. Higher mortality was associated with age greater than 50 years (p=0.007), the presence of malignancy (p=0.013), colonic perforation (p=0.014), and fecal contamination (p=0.004). There was no significant correlation with gender (p=0.81), the presence of organ failure (p=1.6), delayed presentation, i.e., preoperative duration >24 hours (p=0.17), and the presence of diffuse peritonitis (p=0.25).

Conclusion

MPI is a specific, easily reproducible, and less cumbersome scoring method for predicting mortality in patients with hollow viscus perforation (secondary) peritonitis with minimal laboratory investigations. Higher scores correlate with a poorer prognosis and need intensive management, making use of MPI in clinical practice relevant and beneficial, especially in resource-poor settings.

## Introduction

Acute onset diffuse secondary peritonitis due to hollow visceral perforation is a serious life-threatening condition associated with significant morbidity and mortality. Treatment is primarily surgical, and early intervention is always desired in case of doubt. Despite significant advances in diagnostic modalities, surgical treatment, and intensive care, the prognosis of such patients remains poor. The lack of surgical facilities, intensive care units, and higher antibiotics in rural setups worsen the outcomes. Early identification and stratification of patients with severe peritonitis may help in selecting patients for aggressive surgical management and selective intensive care approach, especially in resource-poor countries like India. The etiological factors and perforation site for secondary peritonitis differ in Western developed and developing countries [1-3]. Various severity scoring systems have been developed utilizing clinical, laboratory, and imaging both in preoperative and perioperative settings like the Mannheim peritonitis index (MPI), Peritonitis index of Altoma, physiological and operative severity score for the enumeration of mortality and morbidity, acute physiological and chronic health evaluation, and sepsis severity score [4]. Most of these scoring systems are tedious to measure, require the latest diagnostic investigations not easily available in resource-poor countries, and need multiple measurements of numerous factors. Also, all the scoring systems developed are from Western countries and need to be analyzed in the context of different populations from developing countries before generalizing their utility. Recent well-established scoring systems do not consider intraoperative findings, such as the nature of exudate/contamination, and the source or level of perforation which alter the outcome of such cases [5,6]. The degree of surgical facilities available in rural and urban areas of developing countries also varies. Wacha and Linder [7] developed the MPI after identifying 8 out of the 17 risk factors with prognostic relevance using discriminant analysis and are currently employed widely for predicting mortality from perforation [8]. Multiple studies have confirmed its usefulness, mostly from Western literature and the urban areas of developing countries. This study was conducted to evaluate the effectiveness and accuracy of the MPI in a resource-poor rural setting of a low-middle-income country like India and to estimate the mortality and morbidity based on the MPI.

## Materials and methods

This prospective observational analytical study was conducted with 50 patients at Acharya Vinoba Bhave Rural Hospital from July 2016 to July 2020. After obtaining approval from the Institutional Ethics Committee (DMIMS(DU)/IEC/2015-16/2057), the consenting patients with hollow viscus perforation with secondary peritonitis based on clinical and imaging findings were included in this study. Patients who were below 15 years, those with trauma and primary peritonitis, and those who refused surgery or were medically unfit were excluded from this study. Preoperative laboratory investigations, including complete blood count, liver function tests, renal function tests, chest X-ray with an erect abdominal radiograph, and ECG, were conducted at the time of admission. The patients were resuscitated and taken up for emergency laparotomy. Intraoperative findings were noted. The MPI was calculated for each patient based on preoperative clinical, laboratory, and intraoperative findings (Table 1).

**Table 1:** MPI *Definition of organ failure. Kidney: creatinine >1.6 mg/dl, urea >60 mg/dl, oliguria <20 ml/hour; Lung: pO2 <50 mm Hg, pCO2 >50 mm Hg; Shock: hypodynamic or hyperdynamic; Intestinal obstruction: paralysis >24 hrs or complete mechanical ileus. MPI scores of patients were categorized into three groups: (I) score ≤21, (II) score 21-29, and (III) score >29 [9,10].

Sr no	Risk factor	Weightage
1	Age >50	5
2	Female sex	5
3	Organ failure*	7
4	Malignancy	4
5	Preoperative duration	4
6	Origin of sepsis not colonic	4
7	Diffuse generalize peritonitis	6
8	Clear exudate	0
	Cloudy exudate	6
	Fecal exudate	12
	Total score	47

Further postoperative management ICU care was given as necessary as per the Declaration of Helsinki. The patients were monitored postoperatively for primary and secondary outcomes. The primary outcome was to determine and correlate the mortality for each category of the MPI (<21, 21-29, and >29) and the secondary outcome was to correlate the individual clinical parameter components of the MPI with the mortality. The categorical variables are expressed as absolute numbers and percentages. Statistical analysis was done using the chi-squared test to compare the association of the MPI score with mortality and various clinical parameters of the MPI with mortality using SPSS Statistics version 29 (IBM Corp. Released 2022. IBM SPSS Statistics for Windows, Version 29.0. Armonk, NY: IBM Corp). The results with a p-value of <0.05 were considered statistically significant, and <0.001 was highly significant.

## Results

A total of 50 patients who attended the casualty and were diagnosed with secondary peritonitis due to hollow viscus perforation were included in this study. There were two-thirds males (n=33) and one-third females (n=17). The most frequent site of perforation was the appendix (35%), followed by the duodenum (25%), small bowel (20%), gastric or stomach (13%), and colon (7%) (Table 2).

**Table 2:** Distribution of sites of perforation

Sites of perforation	Frequency (%)
Appendix	35
Duodenum	25
Small bowel	20
Stomach	13
Colon	7

The majority of the patients, i.e., 58% (29/50), in our study had an MPI score of <21, while 26% (13/50) of the patients had MPI scores between 21 and 29, and 16% (8/50) of the patients had MPI scores of >29. There was 62.5% mortality (5/8 patients) with an MPI score of >29, 37.5% (3/8 patients) mortality in those with MPI scores between 21 and 29, whereas no mortality was found in patients with a score of <21. The study showed that mortality significantly increases with an increase in the MPI score (Table 3).

**Table 3:** Correlation of the MPI score with mortality MPI: Mannheim peritonitis index

MPI score	Outcome		Chi-squared value	p-value
	Survivors	Non-survivors		
<21	29 (69%)	0 (0%)	18.87	<0.001
21-29	10 (23.8%)	3 (37.5%)		
>29	3 (7.14%)	5 (62.5%)		
total	42 (100%)	8 (100%)		

Various postoperative complications seen in the study group were surgical site infections, wound dehiscence, acute kidney injury, pneumonitis, acute respiratory distress syndrome, and shock. The majority of the patients, i.e., 84% (42/50), were discharged uneventfully, and the rest of the patients, i.e., 16% (8/50), expired. Each component of the MPI was individually studied with mortality, and the findings were noted (Table 4).

**Table 4:** Summary of correlation of the MPI components with mortality

Risk factors	Survivors	Non-survivors	p-value
Age (years)			
>50	11	6	0.007*
<50	31	2	
Sex			
Female	14	3	0.81
Male	28	5	
Organ failure			
Yes	2	5	1.6
No	40	3	
Malignancy			
Yes	1	2	0.013*
No	41	6	
Pre-operative duration (more than 24 hrs)			
Yes	34	8	0.17
No	8	0	
Site of perforation			
Colonic	7	5	0.014*
Non-colonic	35	3	
Peritonitis			
Diffuse	36	8	0.25
Localized	6	0	
Character of exudate			
Clear	7	1	0.004*
Cloudy	35	5	
Fecal	0	2	

The patients who were above 50 years had higher mortality rates (6/17, 35.3%) than those who were below 50 years (2/33, 6%), which was highly significant (p=0.007). There was no statistically significant (p=0.81) difference in mortality when compared between females (3/17, 17.6%) and males (5/33, 15.1%). Though there was higher mortality (5/7, 71.4%) in the patients with organ failure at presentation vs no organ failure (3/7, 42.8%), it was not statistically different (p=1.6). The patients with perforation due to associated malignancy had statistically significant (p=0.013) higher rates of mortality (2/3, 66.67%) compared to those with benign etiology (6/47, 12.8%). Delayed presentation of more than 24 hrs before surgery led to an increased mortality risk (8/42, 19% vs 0/8, 0%) but was not found to be statistically significant (p=0.17). This shows that if operated early within 24 hrs, the risks for mortality can be decreased. The presence of colonic perforation had higher mortality rates (5/12, 41.67%) vs non-colonic sites (3/38, 7.9%) and was found to be statistically significant (p=0.014). There was no mortality with localized peritonitis (0/6, 0%) whereas about 18.1% (8/44) mortality in diffuse peritonitis. However, the difference was not statistically significant (p=0.25). There was 100% mortality (2/2) in cases of fecal contamination vs 12.5% mortality in each with clear (1/8) and cloudy contamination (5/40). The difference was very highly significant (p=0.004) for mortality in fecal contamination compared to clear and cloudy contamination.

## Discussion

Perforation peritonitis is one of the common causes of the surgical abdomen. The prognosis of patients with peritonitis along with multi-organ dysfunction remains dismal despite significant improvement in diagnosis and early aggressive medical and surgical management. Early objective categorization of the severity of peritonitis can aid in selecting patients for an aggressive surgical approach. Numerous scoring systems like the sepsis severity score, simplified acute physiological score, Ranson criteria, acute physiological and chronic health evaluation, which considers 12 physiological variables, and MPI were developed for this purpose. MPI utilizes single one-time investigations at presentation along with due acknowledgment of intraoperative findings, which makes it appealing for the proper allocation of scarce resources. In this study, we evaluated the efficacy of MPI as an independent prognostic scoring system in predicting outcomes in secondary peritonitis.

In our study, 17 patients were more than 50 years old, and 33 patients were less than 50 years old. There was a positive significance of age more than 50 years with mortality (p=0.007). Thus, increasing age correlates with higher mortality similar to the study by Sharma et al. [11]. There was a male preponderance with a male-to-female ratio of 2:1 (33 males and 17 females), similar to the study by Mathur et al. [12] and Huttunen et al. [13]. There was no significant correlation with sex (p=0.81). This was in contrast to the study by Sharma et al. that presented a higher mortality associated with the female sex [11]. The most common site of perforation was the appendix (35%), followed by duodenal (25%), small bowel (20%), gastric (13%), and colonic (7%) (Table 2). This was in contrast to the study by Muralidhar et al. that presented that the most common site of perforation was the duodenum followed by the ileum [8].

The majority of the patients, i.e., 58% (29/50), in our study had an MPI score of <21, while 26% of the patients (13/50) had MPI scores between 21 to 29, and 16 % of the patients (8/50 ) had an MPI score of >29. The patients with an MPI score of >29 had a maximum mortality of 62.5%, and those with MPI scores between 21 and 29 had a maximum mortality of 37.5%, whereas no mortality was recorded in patients with a score of <21 (Table 2). The mortality steadily increased with an increase in MPI score, similar to the studies by Barrera et al. [10] and Sharma et al. [13].

There was no significant correlation of mortality with parameters like organ failure (p=1.6), preoperative duration (p=0.17), or the extent of peritonitis (p=0.25). This is in contrast to the studies by Sharma et al. [11] and Bohnen et al. [14]. There was a significant positive correlation between the site of perforation with higher mortality with colonic perforation compared with non-colonic perforation (p=0.013). This can be due to greater fecal contamination with mixed infections in colonic perforation. This is similar to the study by Muralidhar et al. [8].

There was a higher mortality in the malignant etiology of perforation. This can be due to the elderly population, cancer cachexia, and malnutrition associated with malignancy [15]. The mortality with fecal exudate was 100%, whereas it was 14.2% in clear or cloudy exudate. There was a significant correlation between fecal contamination with mortality compared to clear and cloudy exudate (p=0.004). This is similar to the study by Sharma et al. [11].

Various postoperative complications seen in our study were surgical site infections, wound dehiscence, acute kidney injury, pneumonitis, acute respiratory distress syndrome, and shock in patients with higher MPI scores which correlates with the studies by Budzyński et al. [7].

The majority of the patients, i.e., 84% (42/50), were discharged uneventfully, and the rest of the patients, i.e., 16% (8/50), expired. Different studies have recorded mortality rates ranging from 0% to 6% in the MPI <21 groups, 20% to 28.13% in the MPI 21-29 group, and 50% to 95.65% in the MPI > 29 groups [16-20]. In our study, out of the total mortality of eight patients, 62.5% (5/8) of the patients had an MPI score of >29, 23.1% (3/8) of the patients had an MPI score of 21-29, whereas there was no mortality in patients with an MPI score of 21 (Table 5). This is similar to other studies with increasing mortality with MPI scores.

**Table 5:** Comparison of mortality with MPI score between various studies and the current study

Sr no	Author/s	Study type	Year	Mortality		
				<21	21-29	>29
1	Wacha et al. [16]	Prospective cohort	1987	6	-	50
2	Billing et al. [17]	Meta-analysis	1994	2.3	22.5	59.1
3	Qureshi et al. [18]	Prospective analytical	1999-2001	1.9	21.9	28.1
4	Notash et al. [19]	Prospective analytical	2005	-	-	80
5	Budzynski et al. [9]	Retrospective	2015	1.75	28.13	50
6	Yadav et al. [20]	Prospective analytical	2020	0	20	95.65
7	Current study	Prospective analytical	present	0	37.5	62.5

This study proves that the MPI scoring system is the most simple, cost-effective, and easily reproducible scoring method for assessing morbidity and mortality in secondary peritonitis patients. The higher MPI score helps predict the significant increase in mortality of the patients, thereby allowing for the early planning and judicious use of resources for timely intervention to decrease the mortality.

Limitations

The limitation of MPI is not being able to foresee mortality preoperatively as it is performed using both preoperative and intraoperative findings.

## Conclusions

MPI is a simple, precise, easily applicable, and reproducible prognostic index for predicting mortality in patients with secondary peritonitis due to hollow viscus perforation. Its advantage is its predictive ability with minimal use of laboratory tests and consideration for intraoperative findings, which are not included in most of the well-established and recently used scoring systems. It also helps us in taking timely decisions and interventions regarding critical care unit transfer and in stepping up higher antibiotics. MPI scores should be used routinely in clinical practice especially in low-resource settings as higher scores are significantly associated with a poorer prognosis and mortality.
